# Automatic monitoring of lettuce fresh weight by multi-modal fusion based deep learning

**DOI:** 10.3389/fpls.2022.980581

**Published:** 2022-08-25

**Authors:** Zhixian Lin, Rongmei Fu, Guoqiang Ren, Renhai Zhong, Yibin Ying, Tao Lin

**Affiliations:** ^1^College of Biosystems Engineering and Food Science, Zhejiang University, Hangzhou, China; ^2^Key Laboratory of Intelligent Equipment and Robotics for Agriculture of Zhejiang Province, Hangzhou, China

**Keywords:** growth monitoring, fresh weight, deep learning, lettuce, multi-modal fusion, convolution neural network

## Abstract

Fresh weight is a widely used growth indicator for quantifying crop growth. Traditional fresh weight measurement methods are time-consuming, laborious, and destructive. Non-destructive measurement of crop fresh weight is urgently needed in plant factories with high environment controllability. In this study, we proposed a multi-modal fusion based deep learning model for automatic estimation of lettuce shoot fresh weight by utilizing RGB-D images. The model combined geometric traits from empirical feature extraction and deep neural features from CNN. A lettuce leaf segmentation network based on U-Net was trained for extracting leaf boundary and geometric traits. A multi-branch regression network was performed to estimate fresh weight by fusing color, depth, and geometric features. The leaf segmentation model reported a reliable performance with a mIoU of 0.982 and an accuracy of 0.998. A total of 10 geometric traits were defined to describe the structure of the lettuce canopy from segmented images. The fresh weight estimation results showed that the proposed multi-modal fusion model significantly improved the accuracy of lettuce shoot fresh weight in different growth periods compared with baseline models. The model yielded a root mean square error (RMSE) of 25.3 g and a coefficient of determination (*R*^2^) of 0.938 over the entire lettuce growth period. The experiment results demonstrated that the multi-modal fusion method could improve the fresh weight estimation performance by leveraging the advantages of empirical geometric traits and deep neural features simultaneously.

## Introduction

Plant factories have recently gained vast popularity owing to the advantages of growing efficiently and controlled environment. Plant factory, also known as vertical farm, is an advanced stage of controlled environment agriculture (CEA) that features high yield, high quality, and high efficiency (Graamans et al., 2018; Shamshiri et al., 2018). Compared with traditional agriculture, the internal environmental factors of plant factories can be controlled precisely and automatically. Crop yield and quality are significantly correlated with genetic, environmental factors (physical, chemical, and biological) and cultivation methods during crop growth (Kozai et al., 2019). Growing crops in a plant factory can be regarded as a process of control and optimization, in which crop growth monitoring is a crucial step. Accurate and timely crop growth information can reveal the current growth status and yield potential of crops, which are essential for management decision-making.

Fresh weight is a widely used growth indicator for quantifying crop growth. Crop growth can be defined as a process of increment in biomass or dimensions of a plant (Bakker et al., 1995). The fresh weight is one important quantitative factor that dynamically changes during crop growth. Automatic quantitation of fresh weight can help researchers better understand the crop growth process and the dynamic relationship between crop and environment. For leafy vegetables like lettuce, the fresh weight of plant is composed of the root part and the shoot part. The fresh weight of the shoot part is more directly related to yield as the leaves and stems are harvested as final products. Traditional fresh weight measurement methods are mainly based on destructive sampling, which are time-consuming, laborious, and destructive. Nowadays, most commercial greenhouses and plant factories can grow over 10,000 individual plants per day. Traditional methods by manually operation are facing challenges at this large production scale. Thus, automatic and non-destructive monitoring of crop fresh weight is urgently needed.

Image-based approaches have been widely used in fresh weight monitoring of lettuce (Table 1). Images can provide non-destructive, convenient, and low-cost access to crop growth information (Lin et al., 2022). The main processing steps of image-based approaches include image preprocessing, feature extraction, and fresh weight regression. Constructing an appropriate feature extraction method is the key to improving the model performance. Relating size and shape to weight is a common empirical concept in the field of agriculture (Kashiha et al., 2014; Konovalov et al., 2019). Geometric features extracted from lettuce images can quantitatively describe the characteristic of the canopy, which is helpful in fresh weight estimating. Many image segmentation algorithms are developed to segment plant leaves and backgrounds from RGB images or 3D point clouds and then calculate geometric features such as leaf projection area, volume, and plant height from the segmented data to construct a fresh weight regression model (Jung et al., 2015; Jiang et al., 2018; Mortensen et al., 2018; Reyes-Yanes et al., 2020). These empirical feature extraction approaches show promising results, indicating the low-level features extracted from images have strong correlations with fresh weight.

**TABLE 1:** An overview of existing image-based methods for lettuce fresh weight monitoring.

Method type	Input data types	Sample sizes	Methods	Descriptions	References
Empirical feature extraction	RGB	/	Traditional image processing + quadratic regression	Regression by projected area from top view images	Lee, 2008
	RGB	82	Traditional image processing + linear regression	Regression by pixel counting from top view images	Jung et al., 2015; Jiang et al., 2018
	RGB	/	OpenCV-based segmentation + linear regression	Regression by extracted 2D and 3D geometric features from a stereo-vision system	Yeh et al., 2014; Chen et al., 2016
	3D point clouds	230	Rule-based segmentation + linear regression	Regression by extracted geometric features from colored 3D point clouds.	Mortensen et al., 2018
	RGB	338	Optical flow analysis + gradient boost regression	Regression by extracted leaf movement features from top view images.	Nagano et al., 2019
	RGB	750	CNN segmentation + linear regression	Regression by extracted geometric features from the side and top view images	Reyes-Yanes et al., 2020
End-to-end deep learning	RGB	286	CNN regression	Regression directly by a CNN model	Zhang et al., 2020
	RGB-D	3,888	CNN regression	Regression directly by an RGB-D fusion CNN network	Buxbaum et al., 2022

Deep learning techniques such as deep convolutional neural networks (DCNNs) can extract and learn intricate relationships from data through multiple levels for representation (LeCun et al., 2015). End-to-end deep learning methods based on DCNNs have recently emerged and show opportunities to estimate fresh weight directly from input images (Zhang et al., 2020; Buxbaum et al., 2022). Although most DCNNs are initially designed for classification tasks, they can also perform regression tasks well because of their strong feature learning capability. These end-to-end approaches eliminate the cumbersome efforts of extracting features from plant segmentation results and show potential for the practical application of crop growth monitoring.

Some works demonstrate that combining empirical-constructed and deep neural features is an effective method for image-based applications (Nanni et al., 2017; Kan et al., 2019). The data-driven deep learning methods naturally meet their limits when sufficient training data are unavailable (von Rueden et al., 2021). The low-level features extracted by experience can help to provide complementary information for optimizing the initialization and filtering of proposals for deep learning networks, which leads to higher estimation accuracies.

Multi-modal fusion has received intensive research covering different application domains in recent years. Extracting and combining information from multiple modalities considered for a given learning task can produce an improved performance (Ramachandram and Taylor, 2017). Encouraged by the growing availability of image acquisition devices, RGB-D (Red, Green, Blue – Depth) data has attracted increasing attention in agriculture. As a typical multi-modal data, RGB-D images can provide not only color information but also depth information for each pixel. With the combination of color and depth information, the geometrical traits of targets (size, length, or width) can be accurately measured (Fu et al., 2020), which is more advantageous for describing crop growth status. Fusing color and depth features based on DCNNs is a promising approach for achieving better performance (Eitel et al., 2015; Zeng et al., 2019; Quan et al., 2021). However, existing depth sensors still have some limitations, and depth images are prone to holes on transparent or shiny surfaces (Song et al., 2015), which challenges applying multi-modal fusion for extracting and combining information in practical applications.

In this study, we proposed a multi-modal fusion based deep learning model for automatic monitoring of lettuce shoot fresh weight by utilizing RGB-D images. The model combined geometric traits from empirical feature extraction and deep neural features from CNN. A lettuce leaf segmentation network based on U-Net was trained for extracting leaf boundary and geometric traits. A multi-branch regression network was performed to estimate fresh weight by fusing color, depth, and geometric features. Specifically, the objectives of the study were to (1) achieve an accurate and automatic fresh weight estimation of lettuce by a multi-modal fusion based deep learning model; (2) quantify the benefits of combining geometric traits with deep neural features in improving the model performance; (3) investigate the performance variances of fresh weight estimation for different lettuce varieties in the entire growth period.

## Materials and methods

### Dataset

**FIGURE 1:**
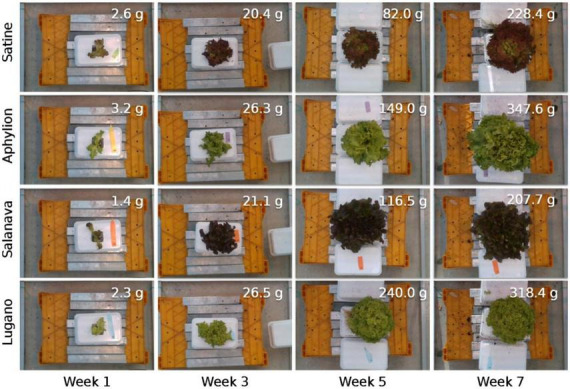
Examples of RGB images (cropped) of the AGC dataset.

The 3rd Autonomous Greenhouse Challenge: Online Challenge Lettuce Images dataset (AGC dataset) was used in this study (Hemming et al., 2021). The dataset was generated for the needs of the 3rd International Autonomous Greenhouse Challenge, a famous international competition held by Wageningen University & Research. Top-view RGB images and aligned depth images of 388 lettuces were provided in the dataset. The dataset contains references to images and measured data on a lettuce crop growing in well-controlled greenhouse conditions. The sampled plants include four different lettuce varieties suitable for hydroponics: Aphylion, Salanova, Satine, and Lugano. Five crop traits were destructively measured at 7-day intervals, including fresh weight of shoot, height, diameter, leaf area, and dry weight of shoot. A total of seven batches of data were collected, covering the entire growth period of lettuce (Figure 1). The AGC dataset did not provide ground truth annotation for leaf segmentation. The CVAT tool (Sekachev et al., 2020) was used to label the leaves and background pixels from RGB images manually.

#### Data preprocessing and augmentation

The original top-view RGB and depth images of the AGC dataset were captured with an image size of 1,920 × 1,080 pixels. We applied the same preprocessing strategy for RGB and depth images of each sample. First, all images were cropped to 1,080 × 720 with the same position parameters (the *x* and *y* coordinate of the upper-left corner is 515 and 210) to remove invalid pixels at the edges. The cropped image dataset was used as the input of the proposed method. We used 70% of the image dataset for training and the rest for testing.

The proposed method consists of two independent networks, which used the same training set and test set, but their data preprocessing and data augmentation methods were different. For the segmentation network, each image is resized to 960 × 640 before being fed to the network. The Albumentations tool was used for data augmentation (Buslaev et al., 2020). Image rotation, rescale, flip, shift, brightness change, contrast change, and RGB change were used in this study. The input images of the segmentation network were augmented one-to-one during training without duplication. For the regression network, the original images of the cropped dataset were used. Since the image scale significantly affects the estimation of fresh weight, only spatial level transforms that maintain the image scale was applied. As a result, an augmented training set containing 2,439 images was constructed before network training.

### Multi-modal fusion model

#### System pipeline for the model

The multi-modal fusion model composed of a lettuce segmentation network and a multi-branch regression network was developed for automatic estimation of the fresh weight of lettuce. The system pipeline of the proposed model included the following steps: (i) data preprocessing, (ii) geometric traits extraction, and (iii) fresh weight estimation. The model’s input was the top-view RGB image, depth image, and empirical geometric traits of lettuce, and the output was the estimation of shoot fresh weight. In the model-building stage, we trained the leaf segmentation network and multi-branch regression network with the same dataset.

#### Leaf segmentation network

**FIGURE 2:**
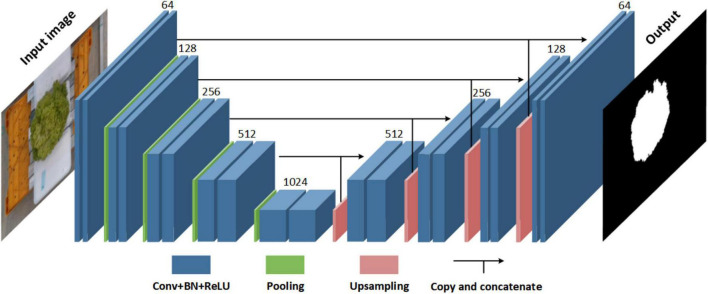
The structure of the U-Net based leaf segmentation network.

A leaf segmentation network based on U-Net architecture was employed to automatically segment lettuce leaves and backgrounds from RGB images (Figure 2). U-Net is a semantic segmentation network based on fully convolutional networks with a typical U-shaped encoder-decoder architecture (Ronneberger et al., 2015). The U-Net architecture used in this study consists of two parts: contractive path (encoder) and expanding path (decoder). The contractive path can extract feature maps of different resolutions by stacking convolutional layers and max-pooling layers, thereby capturing both global and local features of the input images. The expanding path combines features and spatial information at each level by a sequence of up-sampling and concatenation.

As a pixel-wise classification network, each pixel in the input image of the leaf segmentation network corresponds to one instance, where leaf pixels are labeled as 1, and background pixels are labeled as 0. The input image size is 960 × 640 × 3 to balance further processing requirements and the memory limitation. The number of levels, resolutions and channels of each feature map is shown in Figure 2. The Dice loss was used as the loss function (Milletari et al., 2016). The 2-class variant of the Dice loss function was calculated as follows (Equation 1):


(1) $$L\,o\,s\,s_{d\,i\,c\,e}=1-\frac{2\,\sum_{i}^{N}p_{i}\,g_{i}}{\sum_{i}^{N}p_{i}^{2}+\sum_{i}^{N}g_{i}^{2}}$$

where *N* is the sum of pixels, *p*_*i*_ and *g*_*i*_ are the ground truth and prediction label at pixel *i*, *p*_*i*_,*g*_*i*_∈[0,1].

To quantitatively evaluate the performance of the leaf segmentation model, different metrics were used, including intersection over union (IoU), F1 score, pixel accuracy, precision, and recall.

#### Geometric traits extraction

A total of 10 geometric traits were defined to describe the structure of the lettuce canopy (Table 2). We used edge and contour detection methods based on OpenCV to extract geometric traits from segmented images, and the processing step were as follows: (1) removing blobs in the binary image by morphological operations, (2) detecting the largest contour, (3) detecting the minimal circumcircle, minimal area rectangle and convex hull of the contour, (4) calculating geometric traits. The size-related geometric traits (PA, PP, CA, CP, PCD, ARW, and ARH) were obtained directly by pixel counting, and the remaining morphology-related geometric traits (PPR, CPR, and CAR) were calculated by size-related geometric traits.

**TABLE 2:** Geometric traits extracted from the segmented images.

Traits type	Traits	Description
Size related	PA	Projected area
	PP	Projected perimeter
	CA	Convex hull area
	CP	Convex hull perimeter
	PCD	Circumcircle diameter of the projected area
	ARW	Width of minimal area rectangle of the projected area
	ARH	Height of minimal area rectangle of the projected area
Morphology-related	PPR	Projected area/projected perimeter
	CPR	Convex hull area/convex hull perimeter
	CAR	Projected area/convex hull area

#### Multi-branch regression network

**FIGURE 3:**
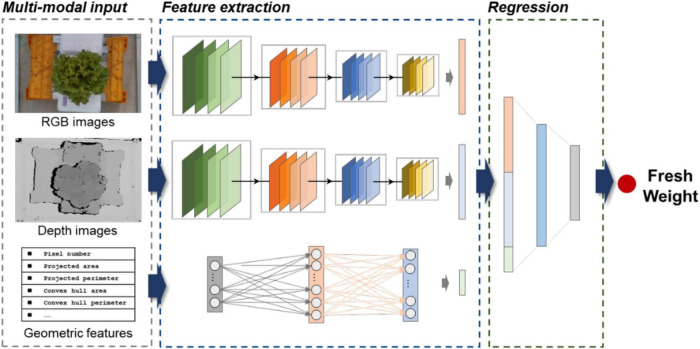
Overall structure of the multi-branch regression network.

A multi-branch architecture regression network was built to fuse multi-modal data for lettuce fresh weight monitoring (Figure 3). The multi-branch regression network was made up of two blocks: feature extraction block and regression block. Min-max normalization was applied for all input variables to speed up the training process. We employed a late fusion architecture to effectively extract and fuse RGB, depth, and geometric features. For RGB and depth branches, we utilized ResNet-34 for feature extraction (He et al., 2015). As a popular network that is widely used in the field of image classification, ResNet can extract deep features from images and have good feature extraction performance. We removed the final 1,000 × 1 fully connected layer of ResNet and obtained the features of RGB and depth from the last flatten layer (average pool). As for geometric features extracted from the leaf segmentation network, we utilized a multilayer perception (MLP) for feature extraction. All outputs of three branches were flattened to ensure they have the same dimensions. Finally, these three feature sets were concatenated and passed to the regression block. The regression block consists of three sequential fully connected layers. In our tests, we found that increasing the depth or width of the regression blocks had little effect on the prediction results. We employed a retraining strategy to train the whole network since the dataset is quite different from ImageNet. The multi-branch regression network used the mean-squared-error (MSE) loss as the loss function (Equation 2).


(2) $$L\,o\,s\,s_{M\,S\,E}=\frac{1}{N}\,\sum_{i}^{N}{\left(y_{i}-{\hat{y}}_{i}\right)}^{2}$$

where *N* is the number of samples, *y*_*i*_ and ${\hat{y}}_{i}$ are the ground truth fresh weight and predicted fresh weight for sample *i*.

Root mean square error (RMSE), mean absolute percentage error (MAPE), and coefficient of determination (*R*^2^) were used as performance indicators for the multi-branch regression network.

### Experimental design and implementation

We performed a model ablation study to comprehensively evaluate the fresh weight estimation capability of the multi-modal fusion model. The multi-modal input of the model consists of RGB images, depth images, and geometric traits extracted from segmented images. It is worthwhile to investigate the performance variances of models with these inputs individually and in different combinations. Specifically, we built three dual-branch baseline models and three single-branch baseline models. The regression block and training configurations of baseline models were as same as the three-branch multi-modal fusion model. Moreover, the fresh weight estimation results of a dataset without data augmentation were presented to evaluate the performance of the model under insufficient data sizes.

All models were trained and tested on a Linux workstation (Ubuntu 16.04 LTS) with two Intel Xeon Gold Processors (2.1G/20 Core/27.5M), 128 GB of RAM, and four NVIDIA GeForce RTX 2080 Ti graphics cards (11 GB of RAM). All the deep learning models were implemented on the Python platform based on PyTorch.

## Results and discussion

### Analysis of the distribution of the lettuce dataset

**FIGURE 4:**
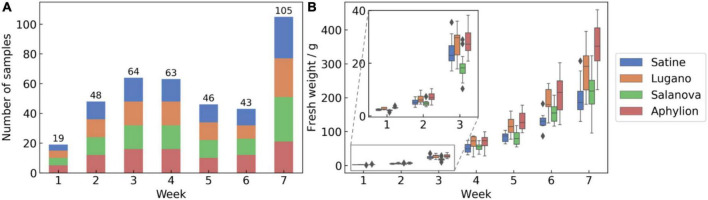
Data distribution of the AGC dataset: **(A)** sample size of the four varieties, and **(B)** fresh weight distribution of the four varieties.

The AGC dataset contains 388 lettuce samples of four varieties growing in 7 weeks. To better describe the dataset, we summarized the data frequency and fresh weight distribution (Figure 4). The sample size of four lettuce varieties was evenly distributed, with a maximum of 102 and a minimum of 92. However, the dataset distribution was skewed toward larger and older plants in terms of the growth period. Moreover, the fresh weight of lettuce varied significantly in different growth periods, ranging from 1.4 to 459.7 g. We also observed that the fresh weight varied across different lettuce varieties, especially in the maturity period. For example, the green leaf varieties (Lugano and Aphylion) were heavier than the red leaf varieties (Satine and Salanova). The unbalanced data distribution potentially challenged the fresh weight estimation of different lettuce varieties in the entire growth period.

### Evaluation of leaf segmentation and geometric traits

**FIGURE 5:**
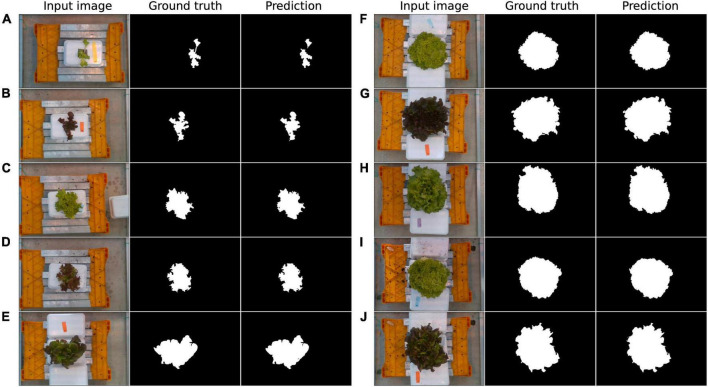
Qualitative results obtained by the leaf segmentation network. 10 samples **(A–J)** were randomly selected.

The leaf segmentation network based on U-Net architecture showed good performance in the AGC dataset (Table 3). We evaluated the pixel-level classification performance of the leaf segmentation network. The leaf segmentation network achieved a mIoU of 0.982 and an accuracy of 0.998 in the test set. For background pixels, all indices were above 99% in the test set. The leaf pixels also obtain satisfactory performance, with an IoU of 0.968 and an F1 score of 0.983 for the test set. The background pixels were more accessible to classify than leaf pixels since background scenes vary less across the dataset. It is noteworthy that the network achieved similarly high results across the test and training sets, indicating that the network was well trained on the dataset without overfitting. Some representative results generated by the leaf segmentation network were also provided (Figure 5). We found the network performed slightly worse in small lettuce through visual appraisal. This may be caused by the relatively small proportion of leaf pixels in the image of small lettuce. The lighter color and loose shape of the leaves also can be factors that influence the performance. In general, the background and leaves were well segmented for lettuce images in different varieties and growth periods, demonstrating the geometric traits extracted from segmented images can accurately describe the morphology of the leaves.

**TABLE 3:** Pixel-level accuracy indices of the leaf segmentation network.

	Class	mIoU	Accuracy	IoU	F1 score	Precision	Recall
Training set	Leaf	0.988	0.998	0.978	0.989	0.989	0.989
	Background			0.998	0.999	0.999	0.999
Test set	Leaf	0.982	0.998	0.968	0.983	0.984	0.983
	Background			0.997	0.999	0.999	0.999

**FIGURE 6:**
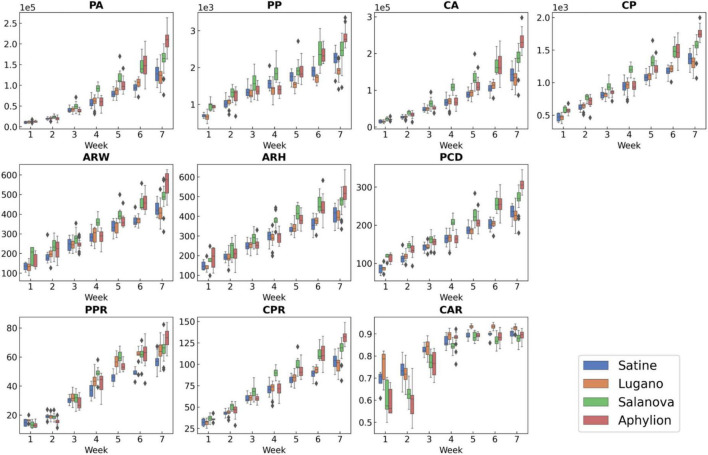
Data distribution of extracted geometric traits of the four lettuce varieties in 7 weeks.

We also analyzed the data distribution of extracted geometric traits of the four lettuce varieties (Figure 6). Both size-related and morphology-related geometric traits showed significant variance in different growth periods. We observed that the canopy size among different varieties had high variance at the maturity period. For example, Aphylion and Salanova had larger canopy sizes than the remaining varieties in weeks 6–7. However, compared with the distribution of fresh weight, Aphylion has a large fresh weight while the fresh weight of Salanova was relatively small. Inconsistencies in canopy size and fresh weight made it difficult for the model to learn comprehensive information for fresh weight estimation at maturity. The correlation between fresh weight and geometric traits of lettuce was analyzed (Table 4). All geometric traits showed significant positive correlations with fresh weight (*P* < 0.001). Such high correlations provide an opportunity for using canopy geometric features to improve fresh weight estimation.

**TABLE 4:** Correlation coefficients between fresh weight and geometric traits of lettuce.

	FW	PA	PP	ARW	ARH	PCD	CA	CP	PPR	CPR	CAR
FW	1										
PA	0.904***	1									
PP	0.799***	0.945***	1								
ARW	0.856***	0.963***	0.944***	1							
ARH	0.838***	0.955***	0.928***	0.896***	1						
PCD	0.862***	0.980***	0.967***	0.968***	0.962***	1					
CA	0.885***	0.997***	0.959***	0.964***	0.955***	0.985***	1				
CP	0.868***	0.986***	0.965***	0.973***	0.970***	0.994***	0.988***	1			
PPR	0.876***	0.933***	0.817***	0.906***	0.919***	0.908***	0.911***	0.932***	1		
CPR	0.873***	0.985***	0.954***	0.969***	0.971***	0.983***	0.982***	0.996***	0.950***	1	
CAR	0.607***	0.627***	0.500***	0.600***	0.632***	0.575***	0.579***	0.622***	0.807***	0.674***	1

FW, fresh weight. ***Correlation is significant at the 0.001 level.

### Fresh weight estimation results

**FIGURE 7:**
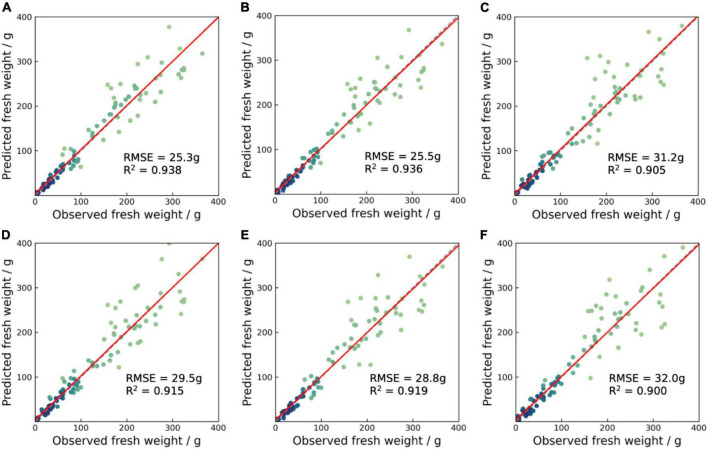
Scatter plots between predicted and observed fresh weight for the test set of augmented models **(A)** RGB + D + G, **(B)** RGB + G, **(C)** D + G, **(D)** RGB + D, **(E)** RGB only, and **(F)** D only. The red solid lines represent the fitting lines, and the gray dotted lines represent the 1:1 lines.

The multi-branch regression network provided a good fresh weight estimation performance for different lettuce varieties in the entire growth period (Table 5). The triple-branch fusion architectures network with RGB, depth and geometric features exhibited the highest estimation performance with an RMSE of 25.3 g, a MAPE of 17.7%, and an *R*^2^ of 0.938. Compared to the dual-branch fusion networks with RGB and depth images, the RMSE was reduced by 4.2 g (14.2%). This improvement mainly came from the late growth period samples (Figure 7). In addition, the models with geometric features showed higher estimation accuracies than the models without geometric features, with a reduction of RMSE by 0.8–4.2 g. The results indicate that fusing the geometric features with deep neural features can lead to a more accurate fresh weight estimation for the image-based deep learning model. Interestingly, the performance of the RGBG fusion model and RGB model was superior to the RGBD fusion model, which was different from prior knowledge. The main reason was that the inevitable noise of depth images made the learning process of the network difficult. Similar results have been found in the field of object detection (Sun et al., 2022). On the other hand, the results implied that the addition of geometric features could help the fusion of multi-modal features.

**TABLE 5:** Fresh weight estimation result of different models in the test set.

	With data augmentation			Without data augmentation		
		
	RMSE/g	MAPE (%)	*R* ^2^	RMSE/g	MAPE (%)	*R* ^2^
RGB + D + G	25.3	17.7	0.938	30.3	21.6	0.910
RGB + G	25.5	18.2	0.936	29.3	23.1	0.916
D + G	31.2	21.2	0.905	35.9	48.2	0.874
RGB + D	29.5	19.6	0.915	36.0	27.8	0.873
RGB only	28.8	17.9	0.919	30.4	39.9	0.910
D only	32.0	25.8	0.900	37.8	44.6	0.861
G only	/	/	/	37.9	34.8	0.860

D, depth; G, geometric features.

We also compared the impact of data augmentation on model performance. All models performed better when data augmentation techniques were applied, with a reduction of RMSE by 1.6–6.5 g. The noticeable improvements demonstrated that data augmentation techniques significantly improved the data generalization and reduced overfitting. We also observed that the models containing depth features were more sensitive to the dataset size. Similar results were found when comparing the performance of models with and without geometric features. The geometric features can improve model performance whether dataset size is limited or sufficient. The model only containing geometric features showed the worst performance among the models, and the performance difference became larger when the model was trained with sufficient data. The deep neural features extracted from images are dominant, however, geometric features still provide complementary information that can help improve the model performance. The reliability of the multi-branch regression network was evaluated. We randomly produced another five different data partitions for training and testing the triple-branch fusion network, with a ratio of 7:3. The triple-branch fusion network showed high performance stability, with a standard deviation of RMSE equals 0.81 g (Supplementary Table 1). The results indicated that the proposed method provides reliable performance for lettuce fresh weight estimation.

### Model performance across different varieties

We compared the model performance of the triple-branch fusion network of each lettuce variety to analyze the influence of varieties (Table 6). The results showed that the varieties with large leaf areas or canopy had poor fresh weight estimation performance. For example, the Satine showed the lowest RMSE of 16.6 g, while Salanova showed the highest RMSE of 30.3 g. As we mentioned in 3.2, Salanova had a large canopy size and a relatively small fresh weight, which affected the estimation performance. The variance in fresh weight distribution and leave color potentially led to inconsistencies in the complexity of model learning in different varieties that affect the model’s generalization ability.

**TABLE 6:** The triple-branch fusion network’s performance of each lettuce varieties.

Variety	RMSE	MAPE (%)	*R* ^2^
Aphylion	29.9	17.4	0.927
Salanova	30.3	24.2	0.899
Lugano	21.3	14.0	0.964
Satine	16.6	15.3	0.956
All	25.3	17.7	0.938

The proposed model with a flexible multi-modal fusion framework showed potential for automatic growth monitoring across different crops in the CEA. Although the model showed high estimation accuracy, many possibilities still remain to be further studied to improve the model, such as its robustness and interpretation capability. For example, the model’s performance in larger lettuce should be further improved. The light environment in plant factories with artificial lighting could be inconsistent with the AGC dataset. It is worthwhile to further perform our model in practical agricultural applications. Enlarging the dataset with more scenarios and applying transfer learning algorithms will effectively improve the model’s robustness. Another limitation is the interpretation capability of the model. Further work could be done to understand and evaluate the contribution of each modality to the performance of the multi-modal fusion model. Adopting model visualization tools such as Grad-CAM (Selvaraju et al., 2017) and SHAPley Additive exPlanations (Lundberg and Lee, 2017) will improve the understanding of the multi-modal fusion model.

## Conclusion

This study proposed a multi-modal fusion based deep learning model for automatic estimation of lettuce shoot fresh weight by combining deep neural features and empirical geometric features. The model is composed of a lettuce segmentation network and a multi-branch regression network. A lettuce leaf segmentation network based on U-Net was trained for extracting leaf boundary and geometric traits. A multi-branch regression network was performed to estimate fresh weight by fusing color, depth, and geometric features. The results demonstrated that: (1) the multi-modal fusion showed good fresh weight estimation performance for different lettuce varieties in the entire growth period. (2) The triple-branch fusion regression network outperformed baseline models, suggesting that the combination of deep neural features and geometric features improves fresh weight estimation performance. (3) The empirical geometric features provided complementary information for improving the model learning ability of lettuce fresh weight estimation, especially for the lettuce with a complex canopy. This study highlights that the fusion of multi-modal data and the combination of deep neural and empirical geometric features are promising approaches for fresh weight estimation of lettuce. The flexible fusion framework can be further applied to the growth monitoring of other crops.

## Data availability statement

Publicly available datasets were analyzed in this study. This data can be found here: https://data.4tu.nl/articles/dataset/3rd_Autonomous_Greenhouse_Challenge_Online_Challenge_Lettuce_Images/15023088.

## Author contributions

ZL, YY, and TL: conception and design of the research. ZL: data preprocessing, model generation and testing, visualization, and writing—original draft. ZL, RZ, and TL: writing—review and editing. ZL, RF, and GR: validation. All authors contributed to manuscript revision, read, and approved the submitted version.
