# Supplementary material for: Automatic monitoring of lettuce fresh weight by multi-modal fusion based deep learning
Source: Front Plant Sci. 2022 Aug 25;13:980581. doi: 10.3389/fpls.2022.980581 (PMC9458202; doi:10.3389/fpls.2022.980581)
Supplement: Supplementary file 1 [file Table_1.DOCX]

Supplementary Material

# Supplementary Figures and Tables

## Supplementary Table

**Supplementary** **Table 1.** Fresh weight estimation results of the triple-branch fusion network by Monte-Carlo cross-validation

|  | RMSE/g | MAPE | R^2^ |
| --- | --- | --- | --- |
| Data partition 1 | 29.3 | 17.3% | 0.934 |
| Data partition 2 | 27.7 | 15.0% | 0.941 |
| Data partition 3 | 28.1 | 14.1% | 0.931 |
| Data partition 4 | 28.6 | 17.9% | 0.928 |
| Data partition 5 | 29.7 | 16.3% | 0.935 |
